# Sports Bra Preferences by Age and Impact of Breast Size on Physical Activity among American Females

**DOI:** 10.3390/ijerph182312732

**Published:** 2021-12-02

**Authors:** Katherine H. Rizzone, Bianca Edison, Nailah Coleman, Cordelia Carter, Ingrid Ichesco, Paige Cassidy, Jane Chung, Courtney Marie Cora Jones

**Affiliations:** 1 Department of Orthopaedics and Pediatrics, University of Rochester Medical Center, Rochester, NY 14624, USA; Courtney_Jones@URMC.Rochester.edu; 2 Department of Orthopaedics, University of South California, Children’s Hospital of Los Angeles, Los Angeles, CA 90027, USA; bedison@chla.usc.edu; 3 Department of Pediatrics, Children’s National Hospital/George Washington University, Washington, DC 20052, USA; ncoleman@childrensnational.org; 4 Department of Orthopaedics, NYU Grossman School of Medicine, New York, NY 10016, USA; cordeliawcarter@gmail.com; 5 Department of Pediatrics, University of Michigan, Ann Arbor, MI 48109, USA; ingridkr@med.umich.edu; 6 Department of Medicine and Pediatrics, Spectrum Health/Helen DeVos Children’s Hospital, Grand Rapids, MI 48197, USA; pacassid2021@gmail.com; 7 Department of Orthopaedics and Pediatrics Frisco, Scottish Rite for Children, University of Texas Southwestern Medical Center, Dallas, TX 75235, USA; jane.chung@tsrh.org; 8 Department of Emergency Medicine, University of Rochester Medical Center, Rochester, NY 14627, USA

**Keywords:** female athlete, sports bra, sporting equipment, physical activity

## Abstract

For many females, sports bras are an essential piece of equipment for participation in sports and physical activity. Breast pain or discomfort from lack of support may be a contributing factor to the noted gender disparity in physical activity of females compared to males. Our objective was to evaluate sports bra usage and characterize sports bra preferences of an active female cohort. This multicenter cross-sectional survey study was conducted at five geographically distinct academic centers. Our measure was administered during outpatient sports medicine clinic visits to females aged 11–64 years old. Chi-square tests were used to compare characteristics across subgroups. Our analysis consisted of 438 respondents, with a mean age of 22 ± 12.2 years. More than a quarter (27.4%) reported lack of breast support prevented them from being active or exercising. Age (*p* = 0.03), breast size (*p* < 0.0001), and household income (*p* = 0.01) were significantly associated with greater frequency of physical activity being limited by lack of breast support. Lack of breast support may be an important barrier for young females of specific populations to meeting physical activity recommendations. Further research is needed to improve the understanding of this important piece of sporting equipment for women.

## 1. Background

Since the passage of Title IX almost 50 years ago, the number of women participating in sports has dramatically increased at the scholastic, collegiate, Olympic, and professional levels [1,2,3,4,5,6]. With increasing female participation and gender parity in a greater variety of sports, the nuanced needs of female athletes, including specific equipment requirements, are becoming part of a larger sports medicine conversation. Typically, when sports equipment is discussed, the focus is on protective gear, such as pads, mouthguards, and helmets, or on the particular equipment necessary for specific sports (i.e., sticks, bats, or shoes). Sports bras are infrequently part of the equipment dialogue. The sports bra needs to be regarded as an essential piece of sporting equipment, as many females need to wear one to be able to participate comfortably in sport and physical activity. Breasts are complex, soft tissue masses of varying densities that lie over deformable muscles that need proper support for various physical activities, hence the importance of proper fit. Breast pain impacts up to 60% of women at rest and increases in frequency when women are active [7]. Larger breast size correlates with greater negative impact on both women’s chest posture at rest [8,9] and increased breast motion during activity [9]. Increased breast motion can alter biomechanics [10,11,12,13], leading to breast and back pain. Additionally, increased breast size and breast motion often have been correlated to decreased self-confidence in women, which may also negatively impact activity levels [14,15].

The first sports bra was constructed in 1977 from two male athletic support structures (jock straps) sewn together by a female costume designer who partnered with two women who had become interested in recreational running but felt limited in their activity level because of lack of breast support [16,17,18]. Prior to its introduction, women wore “regular” or “fashion” bras during activity. Since its initial introduction (the original term utilized was the “Jog bra”), the sports bra industry has grown to more than a $9 billion annual market. It consists of approximately 25% of the total bra market and is estimated to grow to $38.4 billion by 2026 [19]. While sports bras have now existed for decades, there is still a paucity of data specifically examining their characteristics and impact on activity. Early researchers hypothesized about the important components that well-designed sports bra offered (good support, constructed of comfortable and absorptive fabric, supportive straps, padding, and structural components that limited motion, as per Haycock et al., 1978). Additional researchers surveyed female athletes for their desired components and results were similar (price, support, availability of different sizes, comfortable and wicking fabric, and comfortable straps, as per Torgan, 1982, and Gehlsen and Albohm, 1980). In designing an appropriate sports bra, one must consider multiple components: level of support, comfort, fabric, strap orientation, consumer appeal, and price.

Concurrent to the increase in sports participation of many females, the United States has also experienced an increase in the rates of obesity. The World Health Organization 2020 guidelines on physical activity and sedentary behavior recommends that adults participate in 150–300 min of moderate intensity activity per week and adolescents engage in physical activity that averages out to 60 min a day over a week [20]. Very few Americans successfully fulfill this recommendation, and females meet it even less often than their male counterparts [21,22]. Reasons for this are multifactorial, but it has been proposed that breast size may play an important role in this activity disparity [10]. A study of more than 2000 adolescent females in the United Kingdom in 2016 highlighted that larger breast size negatively impacted participation in sports and exercise, [23]. An Australian study of more than 300 adult women reported the same relationship (Coltman et al. 2019).

Our objective sought to examine sports bra preferences among female patients cared for in sports medicine outpatient clinics in the United States and examine how breast size may influence physical activity level. Our group hypothesized that women with larger breast sizes would report a greater frequency of physical activity limitation due to lack of breast support and/or breast pain as compared to those reporting a smaller breast size.

## 2. Methods

This was a cross-sectional survey study conducted at multiple academic medical centers (Ann Arbor, MI, USA; Dallas, TX, USA; Los Angeles, CA, USA; Manhattan, NY, USA; Rochester, NY, USA; and Washington, DC, USA) in the United States from 2019 to 2021. The target population included 11–64-year-old English-speaking, female patients, who were evaluated in a sports medicine clinic by one of the study co-investigators for a musculoskeletal or sports medicine complaint. The one-time, anonymous survey was administered via electronic tablet or paper. A literature review was performed to identify the scientific publications related to sports bra fit and usage, and this previous work was reviewed for important concepts of sports bra preferences and use. The survey was developed in REDCap [24] by the study team and, prior to administration to participants, reviewed by area experts and was piloted in a small sample of the population being recruited. The survey was revised iteratively based off the feedback obtained from these groups. Final survey items consisted of inquiries about the respondents’ preferred sports bra characteristics (strap orientation/width, sports bra fitting experience, price, fabric), breast/bra size (self-reported based on fashion/regular conventional bra sizing), physical activities the respondents participated in, and the respondents’ demographics. Survey item answers included Likert scale, free text response, and multiple choice. Examples of different sports bra characteristics were also included for some of the questions and images were utilized in REDCap as appropriate (i.e., picture of what was meant by an encapsulation bra, compression bra, type of sports bra strap orientation; see the Appendix). Respondents completed the survey while in clinic, which, for most participants, REDCap timestamps showed took less than 10 min. Institutional Review Board (IRB) waiver of documentation of informed consent and assent was obtained from the main IRB site at the University of Rochester Medical Center.

The primary area of interest was participants’ preferences for certain sports bra characteristics. The secondary outcome was the impact of breast size/support on the participants’ physical activity levels. Basic descriptive statistics were calculated to determine the frequency of respondents indicating specific bra preferences. Chi-square tests of association, and, where appropriate, Fisher exact tests, were used to examine differences between participant demographic subgroups and whether their breast size limited participation in physical activity. Age was categorized using the following groupings: 11–14 years old, 15–18, 19–29, and 30 and older. Race was collapsed into white and Black, Indigenous, Person of Color (BIPOC) categories due to small sample sizes. Similarly, household income was also collapsed into fewer categories. Breast size was dichotomized into two groups for ease of comparison. Small breasts were defined as a AA–A–B cup and large breasts were defined as a C cup or larger. For all categorical comparisons, Cramer’s V was also calculated as a measure of the coeffect in the chi-square statistic and the corresponding *p*-value. SAS 9.4 (Cary, NC) was used for all statistical analyses.

## 3. Results

A total of 438 respondents were included in our final analysis. The mean age of our cohort was 22 ± 12.2 years and 5.5% of our cohort identified as Black/African-American and 11.4% as Hispanic/Latina (Table 1). The majority of females reported having a breast size for regular bras as size B (32.0%) or C (24.0%). Breast size/regular bra cup size varied by age (*p* < 0.0001), with adult participants having larger sizes compared to younger participants (Table 2). More than a third (34.9%) of women reported having a professional regular/non-sports bra fitting, but only 3.7% reported ever being professionally fitted for a sports bra. By far, the most important factor for sports bra selection reported by respondents was support (46%), with brand name (12.6%) and previous experience with the bra type/brand (12.1%) as the second and third most common reasons, respectively. Type of sports bra and strap orientation did not vary by age group. Most respondents in our sample preferred a compression type sports bra (45.5%) as compared to an encapsulation type bra (10.4%) or a combination of the two (34.1%). The majority preferred a racerback strap orientation (61.1%) to vertical (6.9%) or cross-back (14.5%).

Middle range adolescents 15–18 years old (28.6%) and young women aged 19–29 (39.2%) more frequently reported lack of support/breast discomfort as preventing them from being physically active or engaging in exercise, as compared to young adolescents age 11–14 (23.8%) and women 30 or older (20.6%) (*p* < 0.0001). Almost 6% reported wearing more than one sports bra when physically active to feel comfortable/supported; this did not vary by age.

Females with a larger cup size (size C–G) were significantly more likely to report breast support concerns preventing them from being physically active or exercising (58.1% compared to 19.1%, *p* < 0.0001) (Table 3). Household income was also significantly associated with breast size impacting physical activity levels, as women in lower income households reported a lack of support, negatively impacting their activity level at higher frequencies than women in households self-reporting incomes greater than $100,000 (*p* = 0.0112). Physical activity limitations from breast size did not vary by race or ethnicity, but did by age, with women 19–29 reporting a limitation most frequently as compared to other age groups (*p* = 0.03).

## 4. Discussion and Limitations

Our study is the first that has examined sports bra preferences in American women, who make up more than half of the population and have been increasing their rates of sports participation for decades. It is also one of the few published studies that has included data from female adolescents. Our results showed a mean breast size of B and C cup size, with nearly 32% and 24% of respondents, respectively, including cup sizes from AA to G. This wide range points to the need for sports bra development to remain inclusive of all sizes. Several females reported the need to wear more than one sports bra at a time to feel comfortable while participating in physical activity, which could be because they are not in an appropriately fitting sports bra. It is important to note that our data may be skewed to represent an athlete population as survey results were obtained from females presenting to sports medicine clinics and may not be generalizable to all females. Sampling the broader female population may yield different mean size results.

The largest sports bra studies to date have been conducted by researchers in England and Australia. One included a survey of 1285 adult marathoners, and the other surveyed more than 400 adult women of the general population ages 20–35 years old [14,25]. Similar to our study, both studies found a large proportion of their cohorts reported ill-fitting sports bras, which, in turn, affected overall physical activity or capabilities within particular activities. In Brown et al., almost one-third of female marathon runners in London experienced rubbing, chafing, and bra slippage, and lost up to 4 cm in their running stride when wearing an ill-fitting bra [25]. Bowles et al. found that 64% of their respondents had breast pain at some time, but this was not found to be related to bra size, and bra size was not associated with physical activity level, which differed from our results.

The importance of a proper sports bra fitting cannot be understated. While just over a third of respondents reported a professional, regular bra fitting in the past, under 4% of our sample had ever had a professional sports bra fitting. Prior studies report up to 85% of women were wearing poorly fitted bras based on the correct bra size determined by professional bra fitting criteria [26]. Optimal fit can help improve activity volume and efficiency, as shown in a study of adolescents who received an educational booklet about bra fit and support [15]. In a 2012 study, English women were found to have statistically significant differences between the bra size they wear and the size determined by a professional fit [27]. Traditional bra sizing was found to overestimate band size and underestimate bra cup size. The larger the breast size, the more inaccurate the sizing [27]. With the expanding diversity of sports bras in relation to the design (encapsulation versus compression versus hybrid), material, strap orientation, strap width, and band, one cannot become stuck or engrained solely on size. In addition, certain sports bras may be more ideal for particular sports or movement patterns. Research has shown that with different movement patterns, such as running and jumping, breast motion can be different, so different sports bras may be needed to provide optimal support for different activities [28,29,30,31,32,33,34,35,36]. A few companies have created guidelines for sports bra fittings [37,38] and there is a web-based fitting program from an academic center well known for its research in this area [39], but hands-on fittings may only offered in certain areas, which can limit access to proper fit and education around optimal fit.

Financial constraints can serve as barriers to both sport participation and, certainly for females, access to a sports bra. The average cost of a sports bra in the United States is $17–70 per bra and our respondents identified price as an important factor in their bra selection criteria. Furthermore, having to wear more than one sports bra to ensure adequate support raises concern for affordability, which could impact physical activity levels. Our data shows that lower household income was associated with a greater likelihood of women reporting that breast size/lack of support prevented them from participating in physical activity and so the intersectionality of economics and activity level must be further studied to highlight areas of future interventions that could be helpful in promoting increases in activity.

This study is the first of its kind in the United States and included multiple geographic sites, but our study also had limitations. This was a cross-sectional study; we do not have information on non-respondents or individuals of the same population who were not given the survey. Further qualitative research may aid in uncovering more specific details about our results. While our sample was fairly diverse, it was still not reflective of the female population of the United States as a whole, which could have impacted the results. Furthermore, we did not include non-English speakers in our cohort, which also limits generalizability and our perspective. It has been shown that Black and Brown women have lower rates of physical activity and are less likely to meet the recommended WHO Guidelines as compared to their white and male counterparts. There are likely many reasons for this, one of which could be lack of access to a comfortable sports bra. This population is also more likely to be overweight or obese, which often adds to soft-tissue burden in the chest area, adding to the discomfort with physical activity and/or to the need for additional support, potentially compounding an already existent sedentary cycle. As the answers were self-reported, there was no objective data collected on breast/bra size nor potentially correlated factors, such as body mass index. We were not able to clarify answers or probe answers further, which would have allowed for the elucidation of more detailed or nuanced data to explain trends or highlight important issues. Our study did not subjectively nor objectively assess the levels of physical activity, as our survey items focused on if physical activity was limited by breast size/discomfort. However, our limited results are consistent with other authors’ results showing that larger breast size negatively impacts physical activity levels (Burbage 2017, Coltman 2019, and Steele 2020).

## 5. Conclusions

Suboptimal breast support likely negatively impacts the level of physical activity and exercise in a number of females, particularly those with larger breast size. This may be an activity limitation that is important to address. Women make up more than half of the United States population, yet this was the first study examining sports bra preferences exclusively in American women, while also being one of the few publications to include data from younger populations. In addition, the vast majority of prior research on this topic has focused on Caucasian women, and participants with small to medium-sized breasts and optimal body mass indices (BMI). These participants are not reflective of the broad diversity of the female population. Studies that include women from diverse racial groups, broad age ranges, and different BMI’s and body types are necessary. Further robust research is needed in this area to improve our understanding of the limitations of breast size on physical activity and exercise and how we can mitigate those impacts, as physical activity and exercise are crucial components in improving the physical, mental, and emotional health of women, and as there is a paucity of literature examining breast size and its impact on physical activity of females.

## Figures and Tables

**Table 1:** Demographic characteristics of the sample.

Variable	All ages %, (N); N = 438	11–14 %, (N); N = 105	15–18 %, (N); N = 147	19–29 %, (N); N = 79	30+ %, (N); N = 107	Chi-sq
White BIPOC	81.0 (355) 19.0 (83)	75.2 (79) 24.8 (26)	74.1 (109) 25.9 (38)	91.1 (72) 8.9 (7)	88.8 (95) 11.2 (12)	0.001
Hispanic/Latina Not Hispanic/Latina	11.4 (50) 88.6 (388)	14.3 (15) 85.7 (90)	12.9 (19) 87.1 (128)	5.1 (4) 94.9 (75)	11.2 (12) 88.8 (95)	NS
Household income <$49,999 $50,000–99,999 >$100,000 Unknown	14.6% (64) 19.6% (86) 41.3% (181) 24.4% (107)	5.7 (6) 21.9 (23) 29.9 (36) 38.1 (40)	10.9 (16) 14.3 (21) 45.6 (67) 29.2 (43)	32.9 (26) 24.1 (19) 22.8 (18) 20.2 (16)	14.9 (16) 21.5 (23) 56.1 (60) 7.5 (8)	<0.0001

**Table 2:** Characteristics of the sport bras preferences of the sample *.

Variable	All ages %, (N) N = 438	11–14 %, (N); N = 105	15–18 %, (N); N = 147	19–29 %, (N); N = 79	30+ %, (N); N = 107	Cramer V	Chi-sq
Small cup size (AA-B) Large cup size (C-G)	78.8 (345) 21.2 (93)	93.3 (98) 6.7 (7)	80.3 (118) 19.7 (29)	63.3 (50) 36.7 (29)	73.8 (79) 26.2 (28)	0.2890	<0.0001
Have you ever had a professional non-sports bra fitting? Yes No	34.9 (53) 65.1 (285)	18.1 (19) 81.9 (86)	34.0 (50) 66.0 (97)	43.0 (34) 57.0 (45)	46.7 (50) 53.3 (57)	0.2240	<0.0001
Have you ever had a professional sports bra fitting? Yes No	3.7 16) 96.3 (422)	2.9 (3) 97.1 (102)	4.8 (7) 95.2 (140)	1.3 (1) 98.7 (78)	4.7 (5) 95.3 (102)	0.0724	NS
Does lack of breast support or breast discomfort ever prevent you from being physically active or exercising? Yes No	27.4 (120) 72.6 (318)	23.8 (25) 76.2 (80)	28.6 (42) 71.4 (105)	39.2 (31) 60.8 (48)	20.6 (22) 79.4 (85)	0.1423	<0.05
How often does lack of support prevent you from exercising or being physically active (N = 318) Never Occasionally Sometimes Most of the time Always	4.2 (5) 59.2 (71) 29.2 (35) 5.0 (6) 2.5 (3)	0 64 (16) 32 (8) 0 4 (1)	7.1 (3) 54.8 (23) 28.6 (12) 7.1 (3) 2.4 (1)	3.2 (1) 58.1 (18) 35.5 (11) 3.2 (1) 0	4.6 (1) 63.6 (!4) 18.2 (4) 9.1 (2) 4.6 (1)	0.1460	NS
What is the most important factor in how you select sports bra to be physical active in? Amount of support Price Style Fabric Purchase the same bra because I know it works for me Other Brand name Strap orientation (N = 422)	46.2 (195) 12.6 (53) 12.1 (51) 11.4 (48) 9.0 (38) 3.6 (15) 2.8 (12) 2.4 (10)	44.0 (44) 5.0 (5) 11.0 (11) 21.0 (21) 8.0 (8) 5.0 (5) 5.0 (5) 1.0 (1)	49.3 (71) 16.0 (23) 16.7 (24) 5.6 (8) 4.9 (7) 2.8 (4) 4.2 (6) 0.7 (1)	43.4 (33) 21.1 (16) 7.9 (6) 10.5 (8) 14.5 (11) 1.3 (1) 0 (0) 1.3 (1)	46.1 (47) 8.8 (9) 9.8 (10) 10.8 (11) 11.9 (12) 4.9 (5) 1.0 (1) 6.9 (7)	0.2079	<0.0001
Do you wear more than one sport bra when physically active in order to feel comfortable/supported? Yes No	5.9 (25) 84.1 (396)	4.0 (4) 96.0 (96)	7.0 (10) 93.0 (133)	7.9 (6) 92.1 (70)	4.9 (5) 95.1 (97)	0.0631	NS
What type of sports bra do you prefer? (n = 422) Encapsulation (separate cup for each breast) Compression (restricts movement by flattening breasts, no separate cups) Combination of encapsulation and compression I don’t have a preference Other	10.4% 45.5% 34.1% 9.2% 0.7%	9.1 (9) 42.4 (42) 30.3 (30) 16.2 (16) 2.0 (2)	11.1 (16) 44.4 (64) 33.3 (48) 11.1 (16) 0	9.2 (7) 52.6 (40) 35.5 (27) 2.6 (2) 0	11.7 (12) 44.7 (46) 37.9 (39) 4.9 (5) 1.0 (1)	0.1192	NS
What type of sports bra strap orientation do you prefer? N = 422) Vertical (like a tank top) Racerback Cross-back I don’t have a preference Other	6.9% 61.1% 14.5% 16.7% 1.0%	6.0 (6) 59,0 (59) 15.0 (15) 18.0 (18) 2.0 (2)	3.5 (5) 60.4 (87) 17.4 (25) 18.1 (26) 0.7 (1)	10.5 (8) 71.1 (54) 5.3 (4) 13.2 (10) 0	9.8 (10) 56.9 (58) 16.7 (17) 15.7 (16) 1 (1)	0.1104	NS
Which of the following do you prefer? (N = 422) Thin straps Wide straps No preference	16.8% 54.7% 28.4%	17.0 (17) 45.0 (45) 38.0 (38)	15.4 (22) 54.6 (78) 30.1 (43)	18.4 (14) 64.5 (49) 17.1 (13)	17.5 (18) 57.3 (59) 25.2 (26)	0.1123	NS

* Sample sizes for each item response are N = 438 unless otherwise noted in the item stem in the leftmost column.

**Table 3:** Breast support and exercise/physical activity limitations.

	Does Lack of Support/Breast Discomfort ever Prevent you from being Physically Active or Exercising? % Answering Yes	Does Lack of Support/Breast Discomfort ever Prevent you from being Physically Active or Exercising? % Answering No	Cramer V	Chi-Square
Age 11–14 Age 15–18 Age 19–29 Age 30+	20.8 35.0 25.8 18.3	79.2 65.0 74.2 81.7	0.2089	0.03
White BIPOC	26.2 32.5	73.8 67.5	−0.0556	NS
Hispanic/Latina Non-Hispanic/Latina	30.0 27.3	70.0 72.7	0.0209	NS
Cup size AA-B C-G	19.1 58.1	80.9 41.9	−0.3570	<0.0001
Household income <$49,999 $50,000–99,999 >$100,000 Unknown	42.2 31.4 21.6 25.2	57.8 68.6 78.4 74.8	0.1592	0.01

## Data Availability

The data presented in this study are available on request from the corresponding author. The data are not publicly available due to privacy of participants.
